# Synergistic Anti-MRSA Activity of Cationic Nanostructured Lipid Carriers in Combination With Oxacillin for Cutaneous Application

**DOI:** 10.3389/fmicb.2018.01493

**Published:** 2018-07-04

**Authors:** Ahmed Alalaiwe, Pei-Wen Wang, Po-Liang Lu, Ya-Ping Chen, Jia-You Fang, Shih-Chun Yang

**Affiliations:** ^1^Department of Pharmaceutics, College of Pharmacy, Prince Sattam Bin Abdulaziz University, Al Kharj, Saudi Arabia; ^2^Department of Medical Research, China Medical University Hospital, China Medical University, Taichung, Taiwan; ^3^Department of Internal Medicine, Kaohsiung Medical University Hospital, Kaohsiung, Taiwan; ^4^College of Medicine, Kaohsiung Medical University, Kaohsiung, Taiwan; ^5^Pharmaceutics Laboratory, Graduate Institute of Natural Products, Chang Gung University, Taoyuan, Taiwan; ^6^Chinese Herbal Medicine Research Team, Healthy Aging Research Center, Chang Gung University, Taoyuan, Taiwan; ^7^Research Center for Industry of Human Ecology and Research Center for Chinese Herbal Medicine, Chang Gung University of Science and Technology, Taoyuan, Taiwan; ^8^Department of Anesthesiology, Chang Gung Memorial Hospital at Linkou, Taoyuan, Taiwan; ^9^Department of Cosmetic Science, Providence University, Taichung, Taiwan

**Keywords:** nanostructured lipid carriers, cationic surfactant, oxacillin, methicillin-resistant *Staphylococcus aureus*, skin

## Abstract

Nanoparticles have become a focus of interest due to their ability as antibacterial agents. The aim of this study was to evaluate the anti-methicillin-resistant *Staphylococcus aureus* (MRSA) activity of cationic nanostructured lipid carriers (NLC) combined with oxacillin against ATCC 33591 and clinical isolate. The cationic resource on the NLC surface was soyaethyl morpholinium ethosulfate (SME). NLC loaded with oxacillin was produced to assess the antibacterial activity and the effectiveness of topical application for treating cutaneous infection. The hydrodynamic diameter and zeta potential of oxacillin-loaded NLC were 177 nm and 19 mV, respectively. When combined with NLC, oxacillin exhibited synergistic MRSA eradication. After NLC encapsulation, the minimum bactericidal concentration (MBC) of oxacillin decreased from 250 to 62.5 μg/ml. The combined NLC and oxacillin reduced the MRSA biofilm thickness from 31.2 to 13.0 μm, which was lower than the effect of NLC (18.2 μm) and antibiotic (25.2 μm) alone. The oxacillin-loaded NLC showed significant reduction in the burden of intracellular MRSA in differentiated THP-1 cells. This reduction was greater than that achieved with individual treatment. The mechanistic study demonstrated the ability of cationic NLC to disrupt the bacterial membrane, leading to protein leakage. The cell surface disintegration also increased oxacillin delivery into the cytoplasm, activating the bactericidal process. Topical NLC treatment of MRSA abscess in the skin decreased the bacterial load by log 4 and improved the skin’s architecture and barrier function. Our results demonstrated that a combination of nanocarriers and an antibiotic could synergistically inhibit MRSA growth.

## Introduction

The growing amount of drug-resistant strains has become a serious health threat, especially the methicillin-resistant *Staphylococcus aureus* (MRSA) (Tong et al., 2015). Some MRSA strains are even resistant to second-line treatment such as vancomycin and doxycycline (Cihalova et al., 2015). The skin is the major organ infected by MRSA (Dréno et al., 2016). Topical application can be an efficient route to administer antibiotics for direct MRSA eradication. Only 6 antibacterial agents have been approved by the USFDA for MRSA management. Since none of these drugs is used for topical treatment (Rodvold and McConeghy, 2014), the development of new anti-MRSA agents for cutaneous use is urgently needed.

In the past 10 years, nanomedicine has become an innovative approach for combating drug-resistant pathogens. The large surface-to-volume ratio, the possibility of surface functionalization, and the capacity for drug entrapment contribute to the efficient antibacterial activity of nanoparticles (Zazo et al., 2016). Among these nanosystems, lipid-based nanoparticles such as liposomes, nanoemulsions, and nanostructured lipid carriers (NLC) are usually employed for carrying antibacterial drugs. In addition to their role as carriers for antibiotics, some cationic surfactants exhibiting antimicrobial impact can be intercalated in the surface of lipid-based nanoparticles; these include amino acid–based surfactants and quaternary ammonium salts (Colomer et al., 2013; Hwang et al., 2013; Tavano et al., 2014). The combination therapy of more than one antibacterial agent can reveal the synergistic activity against MRSA; thus the dose can be reduced to minimize the adverse effects (Henson et al., 2017). Some investigations involve combining nanoparticles and antibacterial agents for the synergistic inhibition of MRSA infection. For instance, Banche et al. (2015) developed chitosan nanodroplets loaded with oxygen for efficiently eradicating MRSA and *Candida albicans* without resultant cytotoxicity on keratinocytes. Argenziano et al. (2017) demonstrated that ultrasound-mediated vancomycin-loaded nanobubbles were more effective than free vancomycin for killing MRSA. In this study, we aimed to investigate the applicability of synergistic MRSA inhibition by cationic nanocarriers in combination with antibiotic for topical delivery. NLC consisting of mixed liquid and crystalline lipids in the nanoparticulate cores were utilized to load cationic surfactant for enhanced anti-MRSA potency. Soyaethyl morpholinium ethosulfate (SME) was chosen as the cationic surfactant because of the low cytotoxicity against mammalian cells such as neutrophils and keratinocytes (Hwang et al., 2015; Yang et al., 2016).

Oxacillin is a β-lactam commonly used to treat complicated skin infections, but it is ineffective against MRSA invasion (Thomsen et al., 2006). We used oxacillin entrapped in NLC for increased effectiveness against MRSA. A panel comprising *S. aureus*, MRSA, and drug-resistant clinical isolate was used as the pathogens to assess the antibacterial activity of the nanosystems. MRSA in the planktonic, biofilm, and intracellular forms was tested in the present study. The encapsulation of oxacillin in lipid nanoparticles may enhance the delivery ability into the biofilm and host cells. To estimate the *in vivo* efficiency of combined NLC and oxacillin, the transepidermal water loss (TEWL), MRSA burden, and histology were evaluated using a BALB/c mouse model with MRSA skin infection.

## Materials and Methods

### Preparation of NLC

The lipid and water phases of NLC were prepared separately. The lipid phase consisted of 2% squalene, 2% hexadecyl palmitate, 1.5% soy phosphatidylcholine (Phospholipon 80H^®^), 1% deoxycholic acid, and 0.4% SME. The water phase consisted of 1.5% Pluronic F68 and double-distilled water. Both phases were heated to 85°C for 15 min. The water phase was added to the lipid phase in the presence of high-shear homogenization (Pro250, Pro Scientific) at 12,000 rpm for 20 min. The mixture was subsequently treated using a probe-type sonicator (VCX600, Sonics and Materials) for 15 min at 35 W. Oxacillin (0.1%) was included in the lipid phase as needed.

### Size and Surface Charge of NLC

The average diameter and zeta potential of NLC with and without oxacillin were determined using dynamic light scattering (Nano ZS90, Malvern). The nanocarriers were diluted by double-distilled water 100-fold before measurement.

### Oxacillin Encapsulation in NLC

The entrapment percentage of oxacillin was determined by utilizing the ultracentrifugation method to separate the incorporated compound from the free form. The NLC was centrifuged at 48,000 × *g* and 4°C for 40 min. The free antibiotic in the supernatant and the encapsulated antibiotic in the precipitate were analyzed by high-performance liquid chromatography to measure the entrapment efficiency.

### Bacterial Strains and the Culture Conditions

*Staphylococcus aureus* (ATCC 6538) and MRSA (ATCC 33591) were obtained from American Type Culture Collection. KM1 was a clinical isolate of MRSA purchased from Kaohsiung Medical University Hospital. The strains were grown in tryptic soy broth (TSB) medium at 37°C and 150 rpm.

### Minimum Bactericidal Concentration (MBC)

A broth with twofold serial dilution method was utilized to measure the MBC. An overnight culture of bacteria was diluted in TSB to achieve OD_600_ of 0.01 (about 5 × 10^6^ CFU/ml). The bacteria population was exposed to several dilutions of oxacillin and/or NLC with TSB and incubated at 37°C for 18 h. Subsequently, the CFU was counted. The MBC was defined as the lowest concentration that killed ≥ 99.9% of the bacteria.

### MRSA Viability Detection by Fluorescence Microscopy

The viability and death of MRSA after oxacillin and/or NLC treatment were monitored using a Live/Dead BacLight^®^ kit (Molecular Probes). The bacterial pellet was obtained by centrifugation at 12,000 rpm for 3 min. The pellet was resuspended in culture medium (1 ml) with oxacillin (125 μg/ml) and/or NLC (equivalent to 500 μg/ml SME). After incubation at 37°C for 2 h, the resulting suspension was stained with the kit for 15 min. The stained sample was analyzed two-dimensionally by fluorescence microscopy (IX81, Olympus).

### Biofilm Detection

The MRSA biofilm was grown in a Cellview^®^ dish by incubating the bacteria (OD_600_ = 0.1) in TSB containing 1% glucose at 37°C for 24 h. The biofilm was treated with 125 μg/ml cetylpyridium chloride (CPC, the positive control), 125 μg/ml oxacillin, NLC (equivalent to 500 μg/ml SME), or NLC + oxacillin for 24 h. The biofilm was then stained using a Live/Dead BacLight^®^ kit for 15 min. The biofilm was gently rinsed with PBS. The three-dimensional structure was visualized by Leica TSC SP2 confocal microscopy. The SYTO9 green color intensity and biofilm thickness in the confocal images were estimated.

### MRSA Morphology Visualization by Transmission Electron Microscopy (TEM)

MRSA was incubated overnight at 37°C in TSB broth. The bacterial suspension was diluted to achieve an OD_600_ of 0.3. The microbes were then fixed in 3% glutaraldehyde in 0.1 M cacodylate buffer for 2 h. After fixation in 1% osmium teroxide for 2 h, followed by dehydration in an ascending series of ethanol concentrations, the samples were embedded in Spurr’s resin. Sections of 70 nm were stained with 4% uranyl acetate and 0.4% lead citrate prior to observation under Hitachi H-7500 TEM.

### Intracellular MRSA Eradication

Differentiated THP-1 were employed as the host cells to examine the activity of oxacillin and NLC in relation to intracellular MRSA. The differentiation of THP-1 into macrophages was carried out at a phorbol 12-myristate 13-acetate concentration of 100 μg/ml. The cell line was infected by MRSA at an MOI of 50 for 20 min. After being washed with PBS, the cells were incubated in the fresh medium supplemented with 125 μg/ml oxacillin and/or NLC (equivalent to 500 μg/ml SME). Triton X-100 (1%) was pipetted into the medium for cell lysis. The lysate of the cell medium was cultured on the agar dish for 18 h to count the CFU. For the confocal imaging of MRSA killing in THP-1, 4′-6-diamidino-2-phenylindole and anti-*S. aureus* antibody/Alexa Fluor^®^ 488 goat anti-mouse IgG were used to stain the THP-1 nucleus and MRSA, respectively. We also stained the THP-1 actin using an anti-α tubulin antibody/microtubule marker (Alexa Fluor^®^ 594) for visualizing the cytoskeleton under confocal microscopy.

### Proteomic Identification

The MRSA was treated using oxacillin and/or NLC for 3 h. After centrifugation, the bacterial pellet was suspended with 0.5 ml double-distilled water. The MRSA was then centrifuged at 10,000 rpm and 4°C for 15 min after 20-min sonication. The total protein content of MRSA was measured using a Bio-Rad protein assay kit with ELISA at 595 nm. The SDS-PAGE analysis was conducted with a 5% stacking gel and a 10% separating gel followed by silver staining. The bands in the protein gel staining were digested by trypsin at 37°C for 24 h. The digested proteins were acidified with 0.5% trichloroacetic acid and then loaded into an AnchorChip^®^ 600/384. A Bruker Ultraflex^®^ spectrometer was employed for MALDI/TOF/TOF identification. The procedure for this analysis was shown in a previous report (Pan et al., 2010).

### Genomic DNA Analysis

MRSA genomic DNA was extracted using a Presto^®^ Mini bacteria kit according to the manufacturer’s instruction. The aliquot of purified genomic DNA (100 ng) was analyzed by electrophoresis on a 0.8% agarose gel.

### Animal

An 8-week-old female BALB/c mouse was purchased from the National Laboratory Animal Center (Taipei, Taiwan). The animal experiment was done in strict accordance with the recommendations in the Guidelines for the Care and Use of Laboratory Animals of Chang Gung University. The protocol was approved by the Committee of Care and Use of Laboratory Animals.

### *In Vivo* MRSA Infection

The mouse’s back hair was shaved. The back was subcutaneously injected with 1 × 10^6^ CFU MRSA in PBS (150 μl). Subsequently, oxacillin and/or NLC with a volume of 0.2 ml was topically administered on the injection area every 24 h for 3 days. The gross and microscopic appearance of the skin surface was monitored each day. A handheld digital magnifier (Mini Scope-V, M&T Optics) was used to visualize the microscopic skin appearance. TEWL was estimated by Tewameter^®^ TM300 (Courage and Khazaka) from 0 to 3 days post-injection of MRSA. At the end of the experiment, the skin was excised for homogenization by NagNA Lyser (Roche) to count the CFU of MRSA in the skin. The treated skin sample was fixed in 10% formalin, buffered in the phosphate saline, and processed for hematoxylin and eosin (H&E) staining. The unstained slices of formalin-fixed paraffin-embedded skin samples were prepared for immunohistochemical (IHC) staining of lymphocyte antigen 6 complex locus G6D (Ly6G), which is the indicator of neutrophil infiltration. The skin section was incubated with anti-mouse Ly6G antibody for 1 h at room temperature and observed under optical microscopy (DMi8, Leica).

### *In Vivo* Cutaneous Irritation

Oxacillin (625 μg/ml) and/or NLC at a volume of 150 μl were immersed in a non-woven cloth (1.5 × 1.5 cm^2^). This cloth was applied to the dorsal skin of the mouse. Tegaderm^®^ film was used to fix the cloth onto the mouse’s back. The bacterial agent was applied daily for 5 consecutive days. After the treatment, the skin area was monitored by a handheld digital magnifier and TEWL.

### Statistical Measurement

The statistical measurement was conducted using GraphPad Prism 5 software. Dual comparisons were made with unpaired Student’s *t*-test. Groups of three or more were analyzed by ANOVA with Tukey or Dunnett posttests. The significance was indicated as ^∗^ for *p* < 0.05, ^∗∗^ for *p* < 0.01, and ^∗∗∗^ for *p* < 0.001 in the figures.

## Results

### Size and Surface Charge of NLC

**FIGURE 1:**
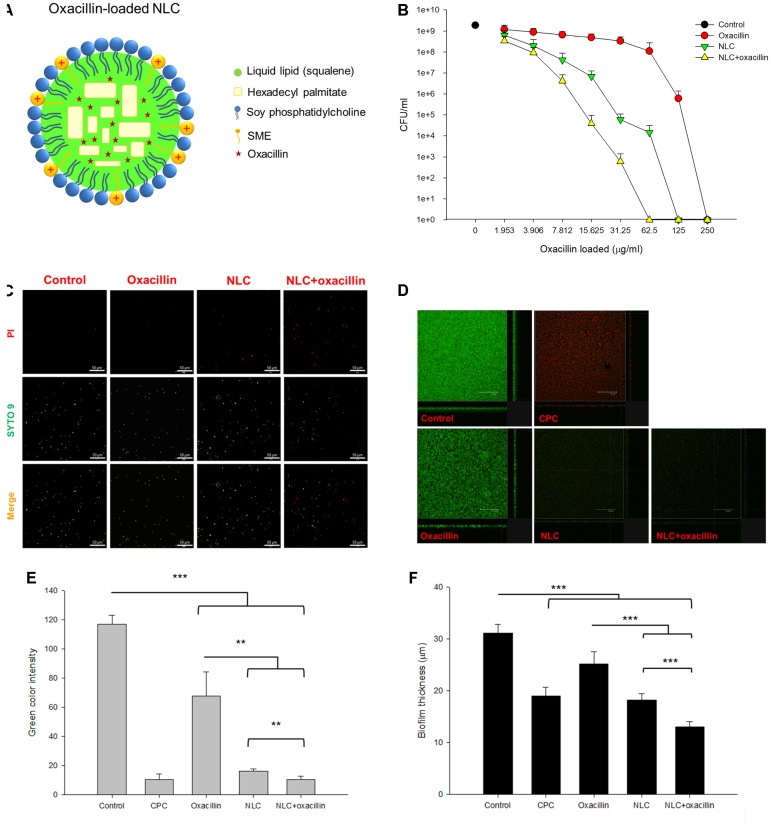
Determination of the anti-MRSA activity of oxacillin and/or NLC. **(A)** The proposed structure of oxacillin-loaded NLC: oxacillin is included in the lipid matrix, whereas SME is intercalated in the emulsifier layers. **(B)** Dose-dependent MRSA killing measured by CFU. **(C)** The planktonic live/dead MRSA strain viewed under fluorescence microscopy. **(D)** The three-dimensional images viewed under confocal microscopy. **(E)** Quantification of fluorescence intensity of MRSA biofilm. **(F)** The corresponding biofilm thickness analyzed by confocal microscopy. Each value represents the mean ± SD (*n* = 3). ^∗∗^*p* < 0.01 and ^∗∗∗^*p* < 0.001.

The molecular structure of oxacillin-loaded NLC is illustrated in **Figure 1A**. We proposed that oxacillin was entrapped in the inner core of NLC, whereas SME was intercalated in the emulsifier layer (oil-water interface). The two antibacterial agents were resided in the different phases of nanoparticulate system. **Table 1** summarizes the diameter, polydispersity index (PDI), and surface charge of the lipid nanocarriers. The average particle size of NLC without the antibiotic was 117 nm, and that of NLC containing oxacillin was 177 nm. Both nanosystems revealed stable unimodal size distribution with PDI of ≤0.3, demonstrating a narrow distribution. Positively charged nanoparticles were achieved (13 mV) for NLC without oxacillin because of the existence of SME on the particulate surface. The oxacillin addition generated greater zeta potential than did the nanoparticles without the drug. The result revealed that the entrapment percentage of oxacillin in NLC was 76.8 ± 7.0%. The encapsulation could be reduced to 59.1 ± 5.5% after 24 h of fresh preparation, indicating a drug release during the experiment.

**Table 1:** The physicochemical properties of NLC and NLC + oxacillin.

Formulation	Size (nm)	PDI	Zeta potential (mV)
NLC	116.92 ± 23.21	0.30 ± 0.02	12.82 ± 2.34
NLC + oxacillin	177.00 ± 9.55	0.29 ± 0.03	18.70 ± 0.82

*Each value represents the mean ± SD (*n* = 3). PDI, polydispersity index*.

### Synergistic Antibacterial Activity of NLC in Combination With Oxacillin

**Table 2** shows the MBC value of oxacillin alone, NLC alone, and the NLC-oxacillin combination. The MBC of oxacillin alone against non-resistant *S. aureus* was 0.488∼0.976 μg/ml, whereas the MBC for SME in NLC was 62.5 μg/ml. The oxacillin MBC was not reduced after NLC incorporation. The combined NLC and oxacillin reduced SME MBC by 16-fold. MRSA was found to be more resistant to NLC and oxacillin as compared to drug-sensitive bacteria. Oxacillin synergized with NLC to inhibit MRSA growth. The oxacillin MBC of treatment alone and in combination with NLC was 250 and 62.5 μg/ml, respectively. The SME MBC of NLC for MRSA decreased twofold after oxacillin entrapment. The clinical MRSA strain (KM1) was more strongly inhibited by NLC and oxacillin than ATCC 33591. The anti-KM1 activity of oxacillin increased in the presence of cationic NLC. The MBC profile clearly indicates an enhancement in antibacterial potency of NLC and oxacillin upon the combination of both agents.

**Table 2:** The MBC of *S. aureus*, MRSA, and KM1 clinical strain after treatment of oxacillin, NLC, and NLC + oxacillin.

Strain	Treatment	Oxacillin (μg/ml)	SME in NLC (μg/ml)
*S. aureus*	Oxacillin	0.488∼0.976	N
	NLC	N	62.5
	NLC + oxacillin	0.976	3.906
MRSA	Oxacillin	250	N
	NLC	N	250∼500
	NLC + oxacillin	62.5	125
KM1	Oxacillin	62.5∼125	N
	NLC	N	62.5
	NLC + oxacillin	7.812	31.25

*N, no data. Each value represents the mean ± SD (*n* = 3)*.

**Figure 1B** represents the concentration-dependent microbicidal action of NLC and oxacillin on MRSA ATCC 33591. The counting of CFU was log-transformed in this figure. No significant decrease of CFU was detected in the oxacillin concentrations of ≤31.25 μg/ml. The oxacillin concentrations of >31.25 μg/ml showed a dose-dependent decrease in viability. With respect to NLC, the inhibitory effect increased with the increased concentration against MRSA. The combination of NLC with oxacillin reduced CFU/ml counts by at least log 5, leading to a killing of >99.9% MRSA at the oxacillin concentration of 62.5 μg/ml. The viability of MRSA was observed with fluorescence microscopy with the staining of dead and live bacteria by propidium iodide (PI) and SYTO9, respectively (**Figure 1C**). The untreated control MRSA was mainly composed of live cells, which were green-stained. Fluorescence analysis revealed that some bacteria co-localized with PI after incubation with NLC and/or oxacillin. Combining nanocarriers and antibiotic proved superior for killing microorganisms as compared to individual treatment because of the increased PI staining.

**Figure 1D** shows the anti-biofilm activity of NLC and/or oxacillin against MRSA under confocal microscopy. The bacteria were able to create a dense biofilm with a large amount of live MRSA after 24 h, which is shown as the negative control. CPC is a cationic surfactant affecting the antibacterial effect via cell wall destruction, with a strong potency. As a positive control, CPC markedly reduced the green signal and enhanced the red signal in the biofilm. Oxacillin exhibited a limited activity against biofilm due to the significant green signal after treatment. The image of biofilm from MRSA treated with NLC alone showed that the biofilm had disintegrated, with an obvious reduction in the number of live bacteria. A similar result was observed in the case of combined NLC and oxacillin. The quantification of green fluorescence in biofilm showed a negligible signal of live cells after CPC treatment (**Figure 1E**). Incubation of oxacillin and NLC alone decreased the green color intensity by about 2- and 6-fold, respectively. The combination permitted a synergistic effect on intensity reduction with statistical significance. Oxacillin-loaded NLC exhibited greater biofilm thickness reduction (13.0 μm) compared to that of drug or NLC alone (**Figure 1F**).

### Intracellular MRSA Killing by NLC and/or Oxacillin

**FIGURE 2:**
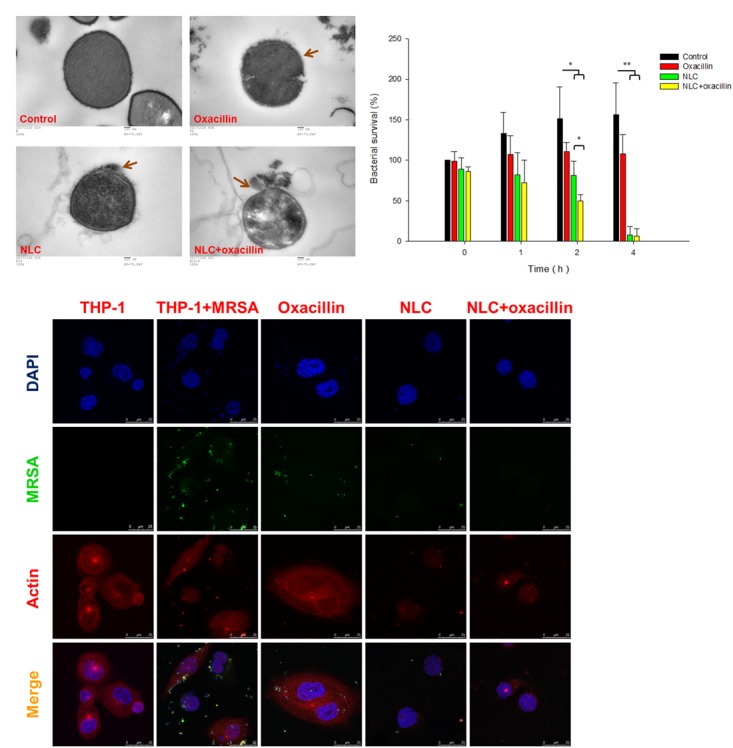
Bacterial morphology change and intracellular MRSA killing by treatment of oxacillin and/or NLC. **(A)** Morphological changes of MRSA viewed under TEM. **(B)** Measurement of MRSA survival in macrophages (THP-1). **(C)** Intracellular MRSA distribution in macrophages (THP-1) viewed under confocal microscopy. Each value represents the mean ± SD (*n* = 3). ^∗∗^*p* < 0.01; ^∗^*p* < 0.05.

**Figure 2A** presents the TEM images of the MRSA morphology. The intact bacteria (control) reveal an integrated cell surface with homogenous cytosol distribution. Oxacillin caused cell membrane deformation with a rough surface (arrow in **Figure 2A**). The MRSA membrane was disrupted after NLC treatment. Some cytoplasmic materials were released from the cytosol (arrow in **Figure 2A**). The observation of fewer dark zones in the cells treated by NLC than in the control can be attributed to cytoplasm dissolution. The cell wall tended to separate from the cytoplasmic membrane since vacuoles formed between them. More cell wall damage and cytoplasmic leakage are seen after combined treatment (arrow in **Figure 2A**). The antibacterial efficacy of oxacillin and NLC was examined using macrophages as the host cells for MRSA infection. Time-dependent intracellular MRSA killing was detected, and is depicted in **Figure 2B**. The MRSA burden in the mammalian cells gradually increased following the increase of time in the untreated control group. Although treatment with NLC and/or oxacillin reduced MRSA production at 1 h, no significant difference was shown after a comparison with the control group. At 2 h, NLC with and without antibiotic resulted in a marked reduction in intracellular MRSA survival. On the other hand, oxacillin alone had no effect on the intracellular viability of bacteria for all the time points tested. Synergy was demonstrated by the combination of NLC and the drug after 2-h incubation. There was a threefold decrease of MRSA survival for the combined treatment as compared with the untreated control. A complete inhibition of MRSA growth occurred consistently after 4 h of contact with NLC alone or NLC + oxacillin.

**Figure 2C** shows the confocal microscopic images of MRSA-infected THP-1 cells. In the images of THP-1 without any treatment, the cytosol is full of red signals, indicating the presence of cytoskeleton stained by actin. Some punctuated green signals in the MRSA-infected THP-1 cytoplasm indicate the invasion of bacteria inside the macrophages. After 4-h incubation of oxacillin or cationic NLC alone, less green fluorescence was visualized in the cytosol. The oxacillin-entrapped NLC resulted in negligible MRSA residence in the cytosol, demonstrating a synergistic effect. We hypothesize that NLC can be utilized as Trojan horses to promote the antibiotic delivery into host cells for killing bacterial.

### Anti-MRSA Mechanisms of NLC and/or Oxacillin

**FIGURE 3:**
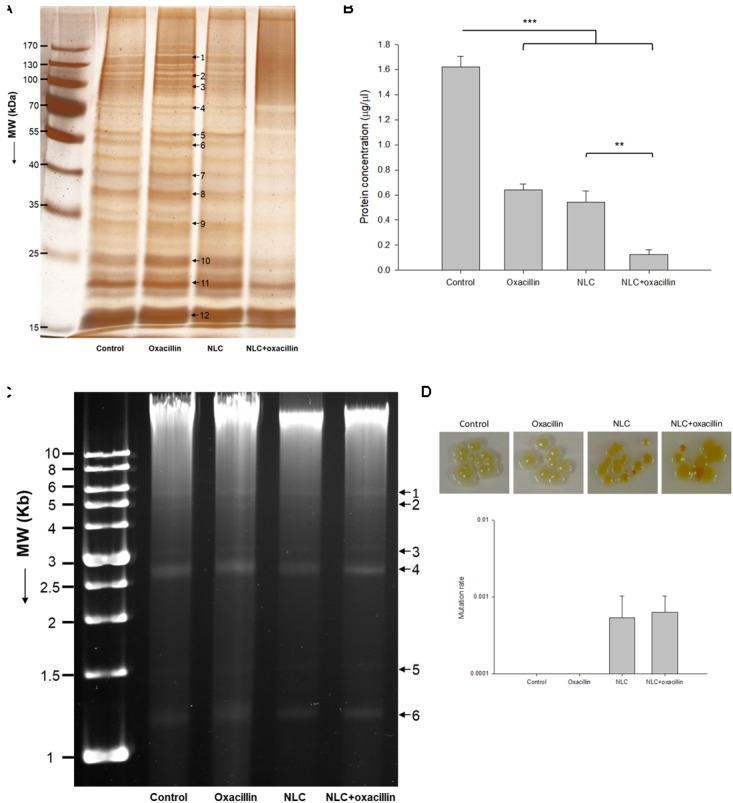
Anti-MRSA mechanisms of oxacillin and/or NLC. **(A)** The protein change of MRSA analyzed by SDS-PAGE and MALDI-TOF/TOF mass. **(B)** Protein concentration in MRSA. **(C)** Analysis of the quality of MRSA genomic DNA by 0.8% agarose gel electrophoresis. **(D)** Mutation rate of MRSA analyzed by colony color change. Each value represents the mean ± SD (*n* = 3). ^∗∗^*p* < 0.01 and ^∗∗∗^*p* < 0.001.

We next explored the anti-MRSA mechanisms of NLC in the presence or absence of oxacillin intervention. **Figure 3A** illustrates the profiles of SDS-PAGE. The bands of NLC- and/or oxacillin-treated MRSA are quite different from those of the untreated microbes. There were 12 protein bands differentially expressed after treatment. The quantification of the protein level was conducted in mass as shown in **Table 3**. All 12 proteins exerted comparable or slightly higher expression by treatment with oxacillin alone, in comparison to the untreated group. No protein exhibited upregulation greater than 2-fold after oxacillin application. The 3 proteins with the highest molecular weights: DNA-directed RNA polymerase subunit β, chaperone ClpB, and elongation factor G, were downregulated by the NLC treatment. Increased expression was detected for the other 9 proteins. However, the expression increment of these 9 proteins was insignificant (<1.25-fold). The NLC and oxacillin combination produced a notable decrease in protein expression, as shown in the SDS-PAGE profiles, especially in the case of ornithine carbamoyltransferase and 30S ribosomal protein S4. Both proteins were decreased by >5-fold in the MRSA with NLC+oxacillin. The total MRSA protein amount was measured after the application of NLC and/or oxacillin as shown in **Figure 3B**. Oxacillin and NLC alone caused a 60 and 66% loss of total protein content compared to the control, respectively. A further reduction was observed with the use of antibiotic-loaded NLC, resulting in a 13-fold decrease.

**Table 3:** Differentially expressed proteins follow the treatment of oxacillin, NLC, and NLC + oxacillin.

Band No.	Protein	Accession No.	MW (Da)	Matched-peptides	Sequence Coverage % (SCORE)	Ratios to control^a,b^			Biological function
						Oxacillin	NLC	NLC + oxacillin	
1	DNA-directed RNA polymerase subunit beta	Q6GBV4	134,735	29	30% (152)	1.82	-0.72	1.32	Initiation factors that promote the attachment of RNA polymerase to specific initiation sites and are then released.
2	Chaperone ClpB	Q6GAV1	87,165	23	41% (167)	1.99	-0.64	1.54	Part of a stress-induced multi- chaperone system, it is involved in the recovery of the cell from heat-induced damage, in cooperation with DnaK, DnaJ and GrpE.
3	Elongation factor G	P68791	76,877	13	22% (113)	1.59	-0.65	1.24	Catalyzes the GTP-dependent ribosomal translocation step during translation elongation.
4	Molecular Chpaerone DnaK	P64408	66,321	21	39% (143)	1.23	0.52	-1.33	Acts as a chaperone.
5	Catalase	Q8NWV5	58,516	17	41% (93)	1.42	1.13	-2.44	Decomposes hydrogen peroxide into water and oxygen; serves to protect cells from the toxic effects of hydrogen peroxide.
6	Enolase	P64079	46,277	17	48% (120)	1.27	0.94	-3.33	Catalyzes the reversible conversion of 2-phosphoglycerate into phosphoenolpyruvate. It is essential for the degradation of carbohydrates via glycolysis.
6	Elongation factor Tu	P64029	43,148	16	58% (109)	1.27	0.94	-2.78	This protein promotes the GTP-dependent binding of aminoacyl-tRNA to the A-site of ribosomes during protein biosynthesis.
7	Arginine deiminase (ARCA)	Q8NUK7	47,139	17	35% (120)	1.33	0.52	-3.85	L-arginine + H_2_O = L-citrulline + NH_3_.
8	Ornithine carbamoyltransferase	Q6GDG8	37,792	14	52% (113)	1.06	0.55	-5.28	Reversibly catalyzes the transfer of the carbamoyl group from carbamoyl phosphate (CP) to the N(epsilon) atom of ornithine (ORN) to produce L-citrulline.
9	Fructose-bisphosphate aldolase class 1	Q6GDJ7	32,875	11	44% (100)	0.96	1	-2.56	D-fructose 1,6-bisphosphate = glycerone phosphate + D-glyceraldehyde 3-phosphate.
10	30S ribosomal protein S4 (RS4)	P66564	23,027	17	61% (159)	1.31	1.25	-6.25	One of the primary rRNA binding proteins, it binds directly to 16S rRNA where it nucleates assembly of the body of the 30S subunit.
11	50S ribosomal protein L6	Q7A084	19,802	10	62% (96)	0.75	0.81	-1.63	This protein binds to the 23S rRNA, and is important in its secondary structure. It is located near the subunit interface in the base of the L7/L12 stalk, and near the tRNA binding site of the peptidyltransferase center.
12	Alkaline shock protein 23 (ASP23)	P0A0P7	19,210	11	68% (124)	0.75	0.80	-0.43	May play a key role in alkaline pH tolerance.

^a^*Ratios to control indicated the fold changes in protein volume among Oxacillin, NLC, NLC + oxacillin treated samples versus MRSA samples, respectively. The higher ratios (>1.0) mean the proteins whose expression levels were increased upon treatments of compounds, while lower ratios (<-1.0) indicate the proteins were downregulated under the exposure to compounds. ^b^Analyzing the gel images using GeneTools software*.

We studied the anti-MRSA mechanisms of oxacillin and NLC by genomic DNA detection, as shown in **Figure 3C**. We differentiated 6 plasmid bands in the agarose gel image. DNA obtained from MRSA treated with NLC and/or the drug exhibited a pattern similar to that found in the control. No significant elimination of DNA was observed by oxacillin or NLC treatment. This result suggests that the extensive damage to the integrity of DNA might not occur when MRSA is treated with both agents; however, small region deletions/insertions or inactivating point mutations of DNA might be occurred. We also examined the possible genomic mutation of the MRSA. The mutation rate assay was performed by spotting a 10-fold dilution of overnight culture onto the agar supplemented with 62.5 μg/ml SME in NLC or NLC + oxacillin. The plate was incubated at 37°C overnight and imaged. As shown in the upper panel of **Figure 3D**, the treatment of NLC alone or the combined strategy was able to modify the color of some colonies from yellow to orange or white, indicating of bacterial mutation. Oxacillin alone did not change the colony color. The mutation rate was calculated based on the MRSA numbers of orange or white colonies normalized to the numbers of total colonies. As shown in the bottom panel of **Figure 3D**, NLC and NLC + oxacillin caused a mutation rate of about 6 × 10^-4^. No colony color phenotype mutation was detected for oxacillin alone.

### *In Vivo* MRSA Infection

**FIGURE 4:**
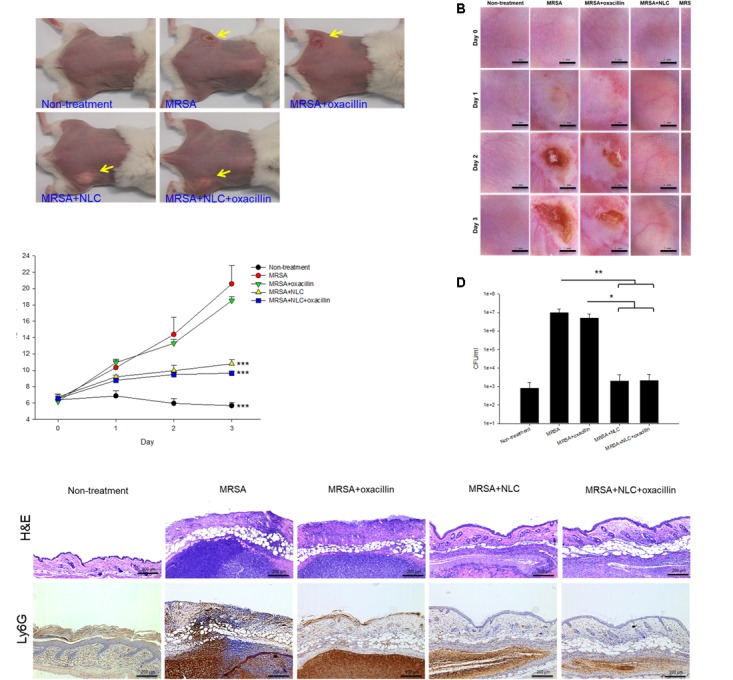
*In vivo* topical application of oxacillin and/or NLC against MRSA. **(A)** The representative macroscopic skin surface observation of mouse after a 3-day treatment MRSA injection. **(B)** The representative skin surface of mouse after treatment of MRSA at day 0, 1, 2, and 3 viewed under handheld digital magnifier. **(C)** TEWL of mice skin after treatment of MRSA at day 0, 1, 2, and 3. **(D)** Survival of MRSA in mice skin treated with MRSA. **(E)** Histological observation of mice skin biopsy stained by H&E and Ly6G antibody after treatment of MRSA. Each value represents the mean ± SD (*n* = 6). ^∗^*p* < 0.05, ^∗∗^*p* < 0.01, and ^∗∗∗^*p* < 0.001.

Nanostructured lipid carriers and/or oxacillin were topically applied onto the region of subcutaneous abscess generated in a mouse model by local MRSA infection. **Figure 4A** shows the demonstrative gross appearance of the mouse back after 3-day treatment. The abscess caused by MRSA is indicated by the arrow in this figure. A significant lesion is seen in the group of MRSA infection without treatment. The improvement in lesion healing was limited in the mouse receiving oxacillin alone. The lesion was reduced with no open wound by topical application of NLC and antibiotic-containing NLC. A nearly complete abscess resolution was detected for the combined NLC and oxacillin. The handheld digital magnifier offered visualization of the changes caused by the MRSA on the demonstrative mouse skin surface, as demonstrated in **Figure 4B**. The end face view of the skin exhibited that the wound was worse following the increase of time in the MRSA and MRSA + oxacillin groups. The treatment with NLC and drug-loaded NLC significantly cleared the abscess with skin texture comparable to healthy skin. The open wound formed by MRSA infection disturbed the cutaneous barrier function. The TEWL-time curves are plotted in **Figure 4C**. The baseline of TEWL (non-treatment) was maintained at 4∼6 g/m^2^/h during 3 days. MRSA inoculation resulted in an immediate and large increase of TEWL, indicating the barrier function deficiency. Oxacillin alone was ineffective in reducing TEWL, while NLC and NLC + oxacillin demonstrated a significant inhibition of water loss in the infected area.

The bacterial count in the skin was estimated 3 days post-injection, as shown in **Figure 4D**. MRSA injection produced a 4-log enhancement in CFU/ml as compared to normal skin. No significant reduction in the MRSA count of oxacillin treatment alone was observed when compared to bacterial infection without intervention. In the mouse infected with MRSA, both NLC and drug-loaded NLC resulted in a 10^4^ reduction of CFU/ml compared with the placebo control. **Figure 4E** shows the qualitative evaluation of skin histopathology of the infection of MRSA with and without intervention. As compared to healthy skin, MRSA injection created a significant disorganization in the epidermis, degenerated dermis, and immune cell infiltration. A large MRSA burden was seen under the subcutis. The generation of the abscess led to thickened tissue. The epidermal damage confirmed the deficiency of barrier integrity as measured by the enhanced TEWL. The wound treated with either NLC or oxacillin-loaded NLC showed a minor inflammation. The distribution of the infiltrated neutrophils in the skin was visualized using Ly6G IHC, as shown in the bottom panel of **Figure 4E**. The large neutrophil infiltration overlapped the MRSA distribution in the subcutaneous region, suggesting deep inflammation. We could also observe the neutrophil diffusion to viable skin. Topical oxacillin suppressed the neutrophil migration in viable skin but not in the subcutaneous area. We found attenuation of neutrophil accumulation after nanoparticle treatment, with the oxacillin-loaded NLC displaying greater amelioration.

### *In Vivo* Cutaneous Irritation

**FIGURE 5:**
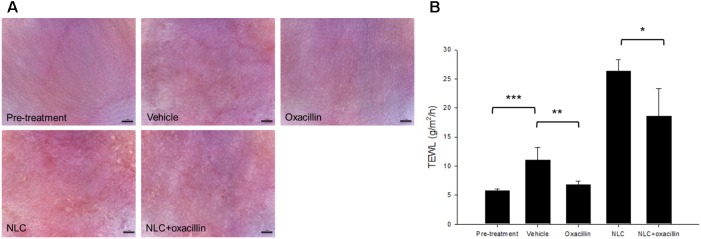
Skin tolerance examination of mouse skin by a 5-day treatment of topically applied oxacillin and/or NLC. **(A)** The representative skin surface of mice viewed under handheld digital magnifier. **(B)** TEWL of mice skin at day 5. Each value represents the mean ± SD (*n* = 6). ^∗^*p* < 0.05, ^∗∗^*p* < 0.01, and ^∗∗∗^*p* < 0.001.

The formulations tested in this study were topically applied on healthy mouse skin each day for 5 days. **Figure 5A** illustrates the representative skin surface images visualized by a handheld magnifier. Slight erythema and scaling occurred when the mouse skin was administered with the vehicle (double-distilled water). In contrast, no visible erythema or edema was observed in the oxacillin-treated skin. The severity of erythema and excoriation was worsened by NLC alone. It was surprising that the addition of oxacillin in NLC could lessen the cutaneous abnormalities caused by NLC. The results of TEWL after 5-day treatment reflected the condition of the skin surface. As shown in **Figure 5B**, the increased TEWL induced by vehicle control was reversed to the non-treatment baseline by oxacillin administration. Application of NLC alone revealed an approximately 4-fold higher TEWL compared to healthy skin. Oxacillin incorporation significantly decreased TEWL from 26 to 18 g/m^2^/h, demonstrating that oxacillin as a protector possesses the capability to reduce possible cutaneous irritation raised by the vehicle or NLC.

## Discussion

Oxacillin is effective in inhibiting non-resistant *S. aureus*, whereas MRSA is known to resist antibiotics such as methicillin and oxacillin (Cihalova et al., 2015). Our results confirm that oxacillin has limited anti-MRSA activity. Oxacillin incorporation in cationic NLC increased the killing efficacy against MRSA. The assembling ability of SME on the nanoparticulate shell was responsible for the antibacterial activity of cationic NLC. The synergistic effect on the antibacterial effect can be described as the combination of two different approaches producing greater activity than either approach alone (Basri and Sandra, 2016). Qin et al. (2013) also define the synergism of two drugs combined causing bacterial killing by a 4-fold lower dose than that of either agent used separately. We achieved the synergism of anti-MRSA activity by combining NLC and antibiotic according to the MBC profile of oxacillin.

Bacterial wall integrity is vital for their survival because it is the outermost and most accessible layer encountering the surrounding environment. It is an important area of action for many antibiotics (Radovic-Moreno et al., 2012). Oxacillin presents cell wall biosynthesis inhibition. The cationic quaternary ammonium surfactants demonstrate antimicrobial activity by targeting and disrupting the cell wall via electrostatic and lipophilic interactions (Zhou et al., 2017). The cell wall of MRSA carries negative charges due to the presence of lipopolysaccharides and teichoic acid (Hemeg, 2017). SME on the NLC surface can bind to the negatively charged cell membrane, permeabilizing it to induce lysis and cellular content leakage. The long alkyl chains in the SME structure might assist the interaction in the cell wall because of the facile intercalation into the lipid bilayers in the membrane. The destabilization of the bacterial wall involves the creation of pores on the cell surface to release ions and molecules as confirmed by our TEM results, after which bacterial death occurs. The demonstration of synergistic antibacterial activity by the combined treatment herein suggests the different mechanisms of action for both antibacterial agents (Bassolé and Juliani, 2012). The oxacillin-loaded NLC interacted strongly with the MRSA surface to damage the membrane. A considerable amount of oxacillin was released from the nanocarriers to generate high local drug concentration near the bacterial surface or inside the bacteria. The sustained bactericidal concentration of combined SME and oxacillin exhibit an anti-MRSA effect superior to that of separate treatment. Oxacillin entrapment increased the positive zeta potential of cationic NLC. The more-positive charges of antibiotic-loaded nanocarriers improved the electrostatic targeting to exert greater MRSA eradication.

Different from the case with planktonic bacteria, conventional antibiotics are less effective in treating biofilm bacteria due to the resistance to antibiotic delivery and avoidance of innate immune intervention (Abee et al., 2011). Using biofilm, we demonstrated that oxacillin-loaded nanoparticles penetrated into the extracellular polymer substance (EPS) and eradicated biofilm MRSA more effectively than individual treatment did. Extracellular DNA plays a key role in biofilm production, acting as a chelator of cationic molecules (Baelo et al., 2015). The interaction between EPS and the nanoparticles featuring lipids can cause a strong affinity and biofilm disintegration (Cheow et al., 2011). The cationic NLC designed in this study fit these criteria. The extremely non-wetting property of biofilm led to the restricted diffusion of antimicrobial liquid formulations (Lin et al., 2017). The low surface tension of cationic NLC caused by the presence of emulsifier systems might assist the penetration into non-wetting biofilm. NLC can hide the physicochemical characteristics of oxacillin to diminish the unfavorable interaction with biofilm.

The killing of intracellular MRSA is a complicated procedure. The pathogens in the host cells favor intracellular replication and the extensive spread of infection. Most of the antibiotics poorly penetrate into the host cells and therefore do not display satisfactory intracellular infection inhibition (Xie et al., 2014). Our results showed that the nanocarriers were preferentially taken up by macrophages, revealing greater activity against intracellular MRSA compared to free oxacillin. The change in the cytoskeleton morphology by the nanoparticles is evidence of phagocytosis (May and Machesky, 2001). The cationic NLC can modify the actin distribution in THP-1 cells; however, it must be noted that NLC might produce some toxicity on macrophages. It is generally recognized that the lipophilic nanoparticles are more facilely phagocytosed into macrophages than are hydrophilic nanoparticles, by the hydrophobic interaction with the cellular membrane (Hsu et al., 2017). The cationic nanoparticles ensure better uptake to macrophages with negatively charged membrane as compared to neutral and anionic ones (Zazo et al., 2016).

Soyaethyl morpholinium ethosulfate on the NLC shell can directly interfere with the bacterial cell wall and damage the membrane. The cationic nanocarriers altered the membrane to release cytoplasmic materials, as shown in the TEM images. The significant loss of proteins in the MRSA co-treated with NLC and oxacillin verified the cell wall leakage; however, the leakage can be acknowledged as mild according to the TEM. The anti-MRSA activity of oxacillin-loaded NLC may also rely on other mechanisms of action. The nanoparticles possibly interact with DNA and the proteins of microbes to disturb the replication, translation, and transcription of the cellular pathways (Hemeg, 2017; Richter et al., 2017). Our previous study (Yang et al., 2016) suggested the antibacterial mechanisms of SME to evoke the Fenton reaction and reactive oxygen species (ROS). The preliminary genome analysis showed no significant alteration of the DNA level after NLC and/or oxacillin management. The greater molecular size of bacterial DNA compared to proteins retarded the leakage to the extracellular space. It is hypothesized that, besides membrane leakage by direct targeting, the nanosystems predominantly acted on proteins, thereby constraining MRSA growth.

The three proteins with the highest molecular weights revealed no significant change under combined NLC and oxacillin. On the other hand, all proteins with the molecular weight of <75 kDa exhibited loss after co-treatment. The mild leakage in the bacterial membrane created by antibiotic-entrapped nanoparticles might allow the liberation of proteins of <75 kDa. Among the detected proteins of >75 kDa, chaperone is central to survival in stress and antibiotic resistance (Frees et al., 2014). It is also a protein predominating in the resistance of MRSA to oxacillin (Jousselin et al., 2012). Treatment of oxacillin and NLC alone moderately increased and decreased chaperone expression, respectively. The combined NLC and oxacillin exhibited an offset effect on chaperone expression. A similar trend was shown in the other two proteins with high molecular weights (DNA-directed RNA polymerase and elongation factor G).

**FIGURE 6:**
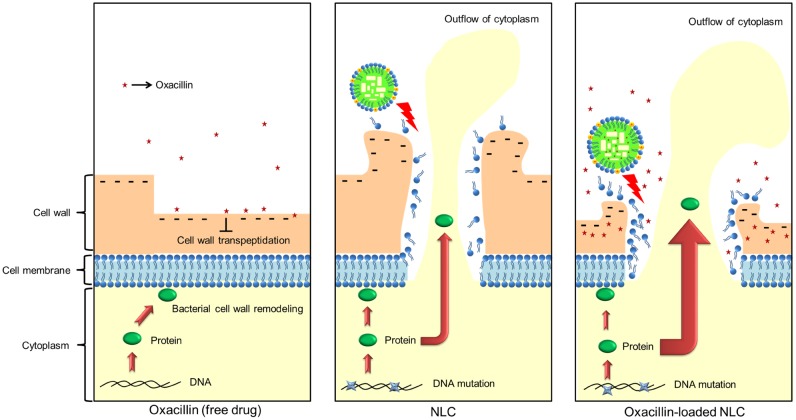
The possible mechanisms of oxacillin and/or NLC for MRSA killing.

Ribosomes, a primary target for some antibiotics, such as macrolides and tetracyclines, are a critical catalyst for substrate stabilization of *S. aureus* protein synthesis (Wall et al., 2015). Oxacillin-loaded nanocarriers might interact and deactivate ribosomal subunits in the same way that some metal nanoparticles do (Hemeg, 2017), which can lead to the obstruction of protein translation. Elongation factor Tu is responsible for the protein synthesis through translation in the ribosomes (Pereira et al., 2015). NLC in combination with the drug showed significant downregulation of both ribosomes and elongation factor Tu. The attachment of invasive phenotype *S. aureus* to the biological surface is a requirement for eliciting virulence infection. Enolase and ornithine carbamoyltransferase are the proteins on the bacterial surface for binding with extracellular matrix proteins such as fibronectin, elastin, and collagen (Hussain et al., 1999; Carneiro et al., 2004). Both surface proteins were largely decreased by NLC and oxacillin co-treatment, thus impeding the pathogenesis of MRSA in the biological system. A similar case was the significant reduction of arginine deiminase after combined treatment. Arginine deiminase is a virulence factor of bacteria in biofilm growth and intracellular survival (Lindgren et al., 2014). The presence of NLC can produce some bacterial mutation. The change of colony color on the agar plate could be due to a response to oxidative stress (Strand et al., 2003), and also indicates of the loss of infectious force. However, it should be noted with caution that the bacterial resistance against the antibiotics may increase after mutation. The oxacillin encapsulation was slightly reduced 24 h post-preparation. Since most of the *in vitro* and *in vivo* experiments were performed within 24 h, we believed that the structure of oxacillin-NLC complex generally remained intact during the experiments. Of course some oxacillin molecules were released from NLC nanoparticles in the nanosystem. It is our opinion that the unencapsulated oxacillin still could synergize with cationic NLC to eradicate MRSA because of the different antibacterial mechanisms of both agents. The possible mechanisms of oxacillin-loaded NLC for killing MRSA are illustrated in **Figure 6**.

The biofilm-like property of bacterial abscess in the skin weakens conventional antibiotic therapy (Han et al., 2009). MRSA contributes to cutaneous inflammation and barrier deterioration. NLC was able to diffuse into the nidus to decrease the MRSA burden and repair the barrier capacity, especially the drug-loaded NLC, which showed smaller abscess size and neutrophil infiltration compared to the NLC without the drug in the *in vivo* experiment. According to the previous studies (Hung et al., 2015), NLC would remain intact because of the soft and deformable characteristics for facile transport into the skin. The fusion of NLC in SC lipids is another possibility (Gelfuso et al., 2016). MRSA infection would damage the skin barrier function because of the formation of wound (Soong et al., 2012; Lin et al., 2017). It was possible that NLC could penetrate into the skin in the intact form. Although NLC can be a potential therapy for MRSA eradication, it is important to examine whether nanotoxicity is induced by the lipid nanocarriers. Our *in vivo* cutaneous tolerance study suggests the symptoms of erythema and excoriation on the skin surface treated by NLC. The TEWL increased 4-fold after 5 consecutive days of topical NLC administration, suggesting barrier disruption. The cutaneous irritation could be classified as mild. A previous study (Shimada et al., 2008) suggests about a 5-fold TEWL increase in dog skin after stratum corneum stripping. Another study (Yan et al., 2010) reports a 10∼25-fold increase of TEWL in rat skin receiving microneedle puncture, which is a permeation-enhancing approach. Both tape stripping and microneedles demonstrate a greater barrier loss as compared to cationic NLC.

It is well-known that quaternary ammonium-based surfactants are typical skin irritants producing some toxicity, including CPC, cetyltrimethylammonium bromide, tri(dodecyldimethylammonioacetoxyl)diethyltriamine trichloride, and benzalkonium chloride (BKC) (Kano and Sugibayashi, 2006; Zhou et al., 2016, 2017). Toxicity is usually a concern in developing antibacterial nanoparticles such as cationic surfactant-coated, zinc oxide, and silver nanoparticles (Pati et al., 2014; Pérez-Díaz et al., 2016). It is critical to improve the safety of cationic nanocarriers while maintaining the antibacterial effect. We had screened a series of cationic surfactants for the cytotoxicity and found that SME demonstrated a wider therapeutic window than the others such as CPC and BKC (Yang et al., 2016). Our previous result approved a safe use of SME. It is the reason why we employed SME in the study.

## Conclusion

We assembled cationic NLC to load oxacillin as a drug-delivery nanosystem for MRSA infection therapy. Since oxacillin encapsulation in NLC achieved > 70%, the free oxacillin was also present in the nanosystems. However, the high loading efficiency of NLC for oxacillin led to the elucidation that the synergistic anti-MRSA effect was mainly attributed to the oxacillin-NLC complex but not free drug or NLC alone. The combined NLC and oxacillin showed lower MBC against MRSA compared to individual treatment. NLC and oxacillin co-treatment increased the efficacy against MRSA residing both extracellularly and intracellularly. The combination was superior in eradicating biofilm compared to mono treatment. Topical administration of oxacillin-loaded nanoparticles significantly reduced cutaneous infection and improved skin barrier function and architecture. NLC has potential for use in combination with antibiotic against MRSA, especially with the currently increasing drug resistance among microbial species. The dose and dosage interval can be reduced with this association. The reduced dose, easy scale-up fabrication, and inexpensiveness of the SME-coated NLC may lessen the expenditure needed for antibacterial therapy. Our nanocarriers can be potential candidates for topical treatment of MRSA infection.

## Author Contributions

AA and J-YF conceived and designed the experiments. P-WW, Y-PC, and S-CY performed the experiments. AA, Y-PC, and S-CY analyzed the data. P-WW and P-LL contributed reagents, materials, and analysis tools. AA, J-YF, and S-CY wrote the paper.

## Conflict of Interest Statement

The authors declare that the research was conducted in the absence of any commercial or financial relationships that could be construed as a potential conflict of interest.
